# Genetic Ablation of CD38 Protects against Western Diet-Induced Exercise Intolerance and Metabolic Inflexibility

**DOI:** 10.1371/journal.pone.0134927

**Published:** 2015-08-19

**Authors:** Shian-Huey Chiang, W. Wallace Harrington, Guizhen Luo, Naphtali O. Milliken, John C. Ulrich, Jing Chen, Deepak K. Rajpal, Ying Qian, Tiffany Carpenter, Rusty Murray, Robert S. Geske, Stephen A. Stimpson, Henning F. Kramer, Curt D. Haffner, J. David Becherer, Frank Preugschat, Andrew N. Billin

**Affiliations:** 1 Muscle Metabolism Discovery Performance Unit, GlaxoSmithKline, Research Triangle Park, North Carolina, 27709, United States of America; 2 QSCI Computational Biology, GlaxoSmithKline, Research Triangle Park, North Carolina, 27709, United States of America; 3 Laboratory Animal Science, GlaxoSmithKline, Research Triangle Park, North Carolina, 27709, United States of America; 4 Target & Pathway Validation, GlaxoSmithKline, Research Triangle Park, North Carolina, 27709, United States of America; Tohoku University, JAPAN

## Abstract

Nicotinamide adenine dinucleotide (NAD^+^) is a key cofactor required for essential metabolic oxidation-reduction reactions. It also regulates various cellular activities, including gene expression, signaling, DNA repair and calcium homeostasis. Intracellular NAD^+^ levels are tightly regulated and often respond rapidly to nutritional and environmental changes. Numerous studies indicate that elevating NAD^+^ may be therapeutically beneficial in the context of numerous diseases. However, the role of NAD^+^ on skeletal muscle exercise performance is poorly understood. CD38, a multi-functional membrane receptor and enzyme, consumes NAD^+^ to generate products such as cyclic-ADP-ribose. CD38 knockout mice show elevated tissue and blood NAD^+^ level. Chronic feeding of high-fat, high-sucrose diet to wild type mice leads to exercise intolerance and reduced metabolic flexibility. Loss of CD38 by genetic mutation protects mice from diet-induced metabolic deficit. These animal model results suggest that elevation of tissue NAD^+^ through genetic ablation of CD38 can profoundly alter energy homeostasis in animals that are maintained on a calorically-excessive Western diet.

## Introduction

Catabolic and anabolic biochemical reactions often require the ordered transfer of electrons and protons between substrates and products. The reduced and oxidized forms of the pyridine dinucleotides, nicotinamide adenine dinucleotide phosphate (NADP^+^) and nicotinamide adenine dinucleotide (NAD^+^) are used as cofactors in these essential oxidation-reduction reactions. Pyridine dinucleotides also regulate various cellular activities, including gene expression, signaling, DNA repair and calcium homeostasis (reviewed in [1, 2]). Relative NAD^+^/NADH ratios also play a crucial role in numerous rate-limiting steps of metabolic pathways (reviewed in [3]). Given the essential role of pyridine dinucleotides in regulating multiple cellular processes, intracellular NAD^+^ levels are tightly regulated and often respond rapidly to nutritional and environmental changes.

For example, NAD^+^ levels increase under conditions of caloric restriction (CR) or fasting [4]. It is thought that this process occurs by inducing nicotinamide phosphoribosyltransferase (Nampt) expression and enhancing NAD^+^ salvage synthesis [5]. In contrast to results observed with CR, a calorically-excessive high-fat Western diet or simple aging can depress tissue NAD^+^ levels (reviewed in [6]). There are at least two plausible approaches to restore depressed NAD^+^ levels to the normal or healthy state. The first approach is to stimulate NAD^+^ salvage synthesis by administration of the NAD+ salvage-pathway precursors, nicotinamide mononucleotide (NMN), nicotinamide riboside (NR) and nicotinamide. Chronic or acute administration of NMN and NR improves body weight, glucose tolerance and insulin secretion in diabetic animal models, suggesting that elevating NAD^+^ levels could provide a therapeutic benefit in human disease [7–9]. The second approach is to inhibit NAD^+^ consuming enzymes, such as CD38, and thereby increase NAD^+^ levels.

CD38 is a multifunctional trans-membrane receptor and enzyme that efficiently hydrolyzes the pyridine dinucleotides NAD^+^, and NADP^+^ [10]. CD38 KO mice have globally elevated tissue NAD^+^ levels and, relative to controls, are protected against diet-induced obesity and metabolic syndrome [11, 12]. Because aging, diet-induced oxidative stress and mitochondrial dysfunction are associated with an NAD^+^ deficit (reviewed in [13]), we were interested in examining the effect of elevating tissue NAD^+^ via genetic ablation of CD38 on the pathogenesis of diet-induced metabolic syndromes and on exercise performance.

In order to study the physiological and functional effects of elevating tissue NAD^+^, it is necessary to develop the working models and to characterize the physiological and physical changes relative to the control. Previous studies have shown that mice fed a high-fat diet (HFD) gain fat mass and have reduced NAD^+^ tissue levels [9, 14]. Another commonly used Western-diet model, the high-fat high-sucrose diet (HFHSD) causes insulin resistance, obesity and increased oxidative stress in skeletal muscle [15–17]. The biochemical effects of a HFHSD on tissue NAD^+^ levels have not been reported in the literature to date. We report here that chronically feeding mice a HFHSD suppresses NAD^+^ levels in tissues and causes a significant deficit in maximal exercise capacity with an associated metabolic syndrome. Mice bearing a genetic deletion of CD38 were protected from diet-induced obesity, hyperglycemia and hyperinsulinemia. Furthermore, CD38 KO mice exhibited a preserved capacity for exercise, a preserved response to exercise and improved metabolic flexibility on HFHSD. These animal model results suggest that elevation of tissue NAD^+^ through genetic ablation of CD38 can profoundly alter energy homeostasis in animals that are maintained on a calorically-excessive Western diet.

## Results

### CD38 KO mice are protected against a HFHS-mediated reduction in tissue NAD^+^ levels

**Fig 1:**
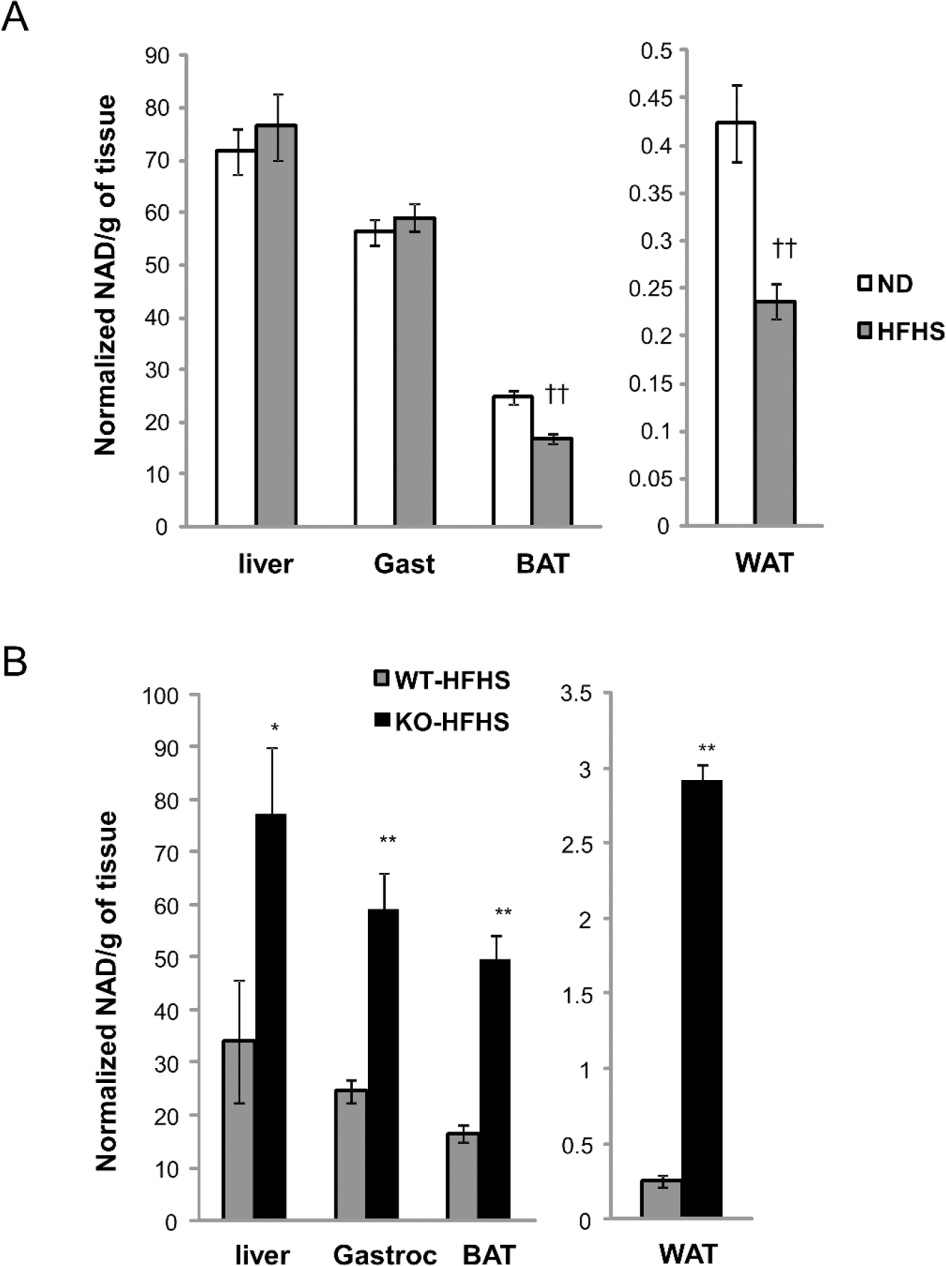
HFHSD reduces tissue NAD^+^ levels and CD38 KO mice have elevated tissue NAD^+^ levels. (A) Tissue NAD^+^ was determined from snap frozen liver, gastrocnemius, brown fat and white fat of WT mice fed a ND (white) or HFHSD (grey) for 5 months. Mice were fasted for 6 hrs before tissue collection. n = 5 per group. ††, p value<0.01 (ND vs HFHSD). See Material and Methods for experimental details. (B) Tissue NAD^+^ was measured from snap frozen liver, gastrocnemius, brown fat and white fat of WT (grey) or CD38 KO (black) mice fed with HFHSD before the study. Mice were fasted for 6 hrs before tissue collection. n = 5 per group. *, p value<0.05 (WT vs KO); **, p value<0.01 (WT vs KO).

We initially compared the effects of normal chow and chronic HFHSD administration on NAD^+^ tissue levels in WT mice. The mice on chronic HFHSD gained substantial body weight mostly due to fat accumulation (Table 1) with a concomitant reduction in NAD^+^ levels in white fat and brown fat (45% and 32% decrease, respectively) (Fig 1A). No significant changes in NAD^+^ levels were observed in liver or the gastrocnemius muscle.

**Table 1:** Parameters of body weight, body composition and serum profile for mice on normal diet or high-fat high-sucrose diet for 4 months

Parameters	Normal diet (ND)	High-fat, high-sucrose diet (HFHSD)
BW (g)	30.5±1.4	46.4±0.7 ^††^
Lean mass (g)	21.5±0.54	22.8±0.23
Fat mass (g)	5.44±0.84	20.0±0.49††
Blood glucose (mg/dL)(fasting)	108.3±5.3	176.2±6.2 ^††^
Serum Insulin (ng/ml)	1.36±0.62	3.11±0.38 ^†^
Serum NEFA (mEq/L)	1.09±0.04	1.43±0.02 ^††^
Serum TRIG (mg/dL)	57±2.88	76.74±1.61 ^††^
Serum Leptin (ng/ml)	2.54±0.53	32.16±1.83 ^††^

N = 10–12 per group.

†, p value<0.05 (ND vs HFHS)

††, p value<0.01 (ND vs HFHSD)

In contrast, CD38 KO mice on HFHSD exhibited significantly higher tissue NAD^+^ levels, including an approximately 2 fold increase in liver, 3-fold increase in the gastrocnemius muscle and brown fat and over 11-fold increase in white fat (Fig 1B). Thus mice fed the HFHSD can respond to the loss of the CD38 gene with increased NAD^+^ levels in multiple tissues, including tissues such as muscle and liver that do not exhibit a diet induced suppression of NAD^+^.

### CD38 KO mice are protected from HFHSD- induced obesity

**Fig 2:**
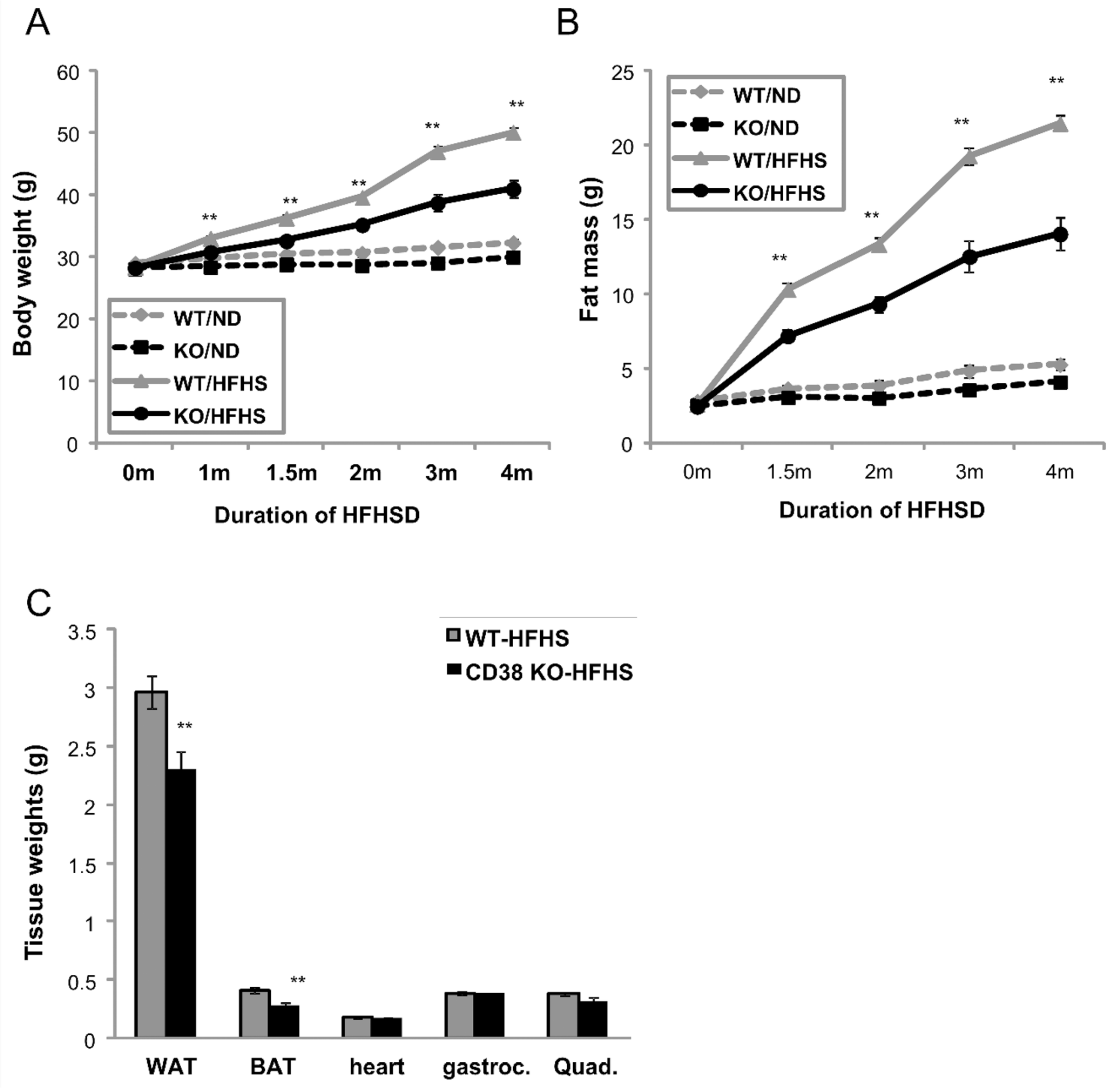
CD38 KO mice are protected from HFHSD- induced obesity. (A) Body weight was measured for WT (grey) and CD38 KO (black) during the 4 months of ND (dashed lines) or HFHSD (solid lines) treatment from age of 2months old. n = 13–15. **, p value<0.01 (WT vs KO). (B) Fat mass was measured by qNMR for WT (grey) and CD38 KO (black) during the 4 months of ND (dashed lines) or HFHSD (solid lines) treatment from age of 2months old. n = 13–15. **, p value<0.01 (WT vs KO). (C) Tissue weights were measured for WT (grey) and CD38 KO (black) after animals were dissected after 4 months of HFHSD. n = 8 **, p value<0.01 (WT vs KO).

**Fig 3:**
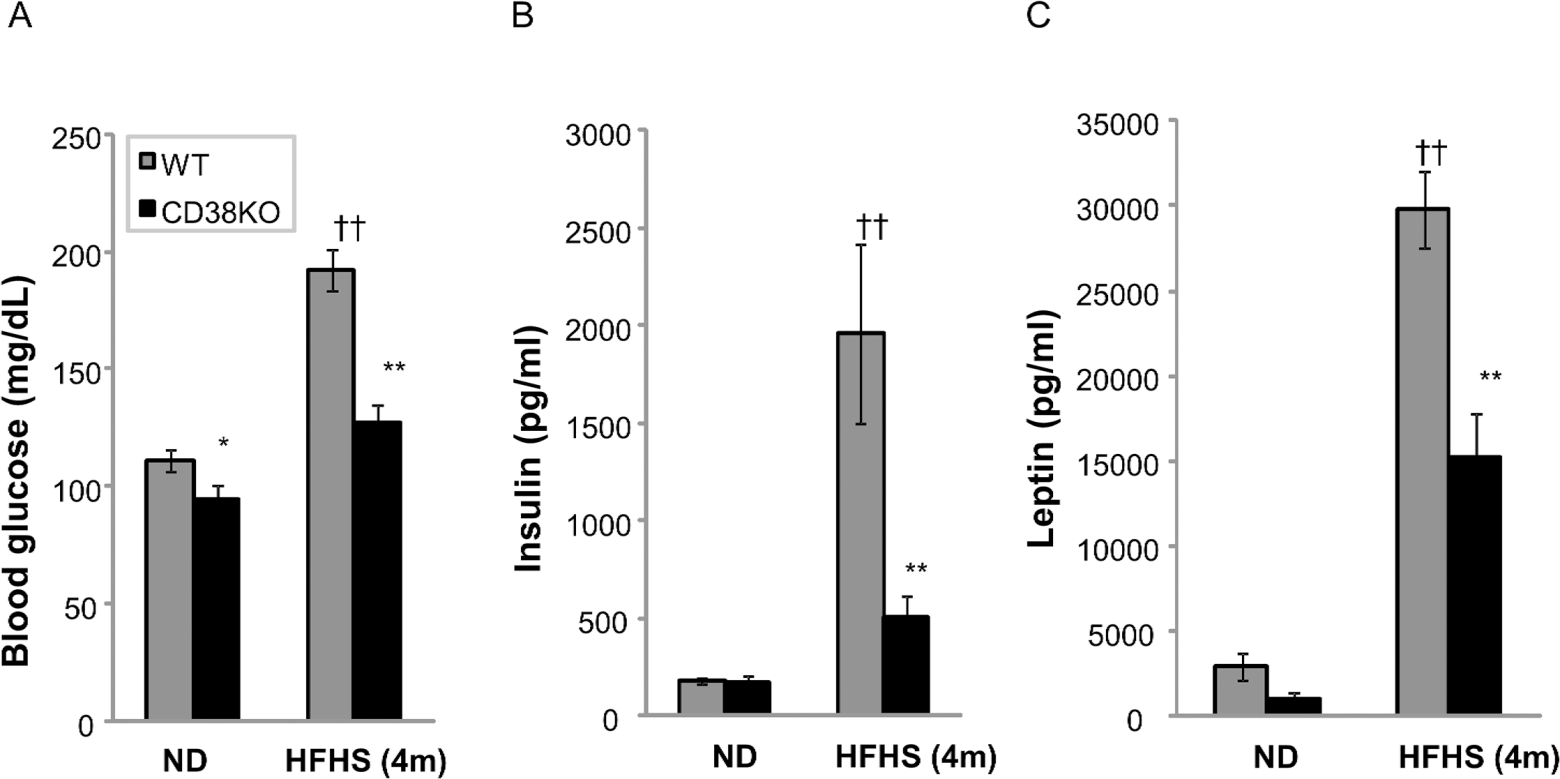
CD38 KO mice are protected from HFHSD- induced hyperglycemia and hyperinsulinemia. (A) Blood glucose was measured for WT (grey) and CD38 KO (black) after 6 hrs of fasting. n = 8. ††, p value<0.01 (ND vs HFHSD); *, p value<0.05 (WT vs KO); **, p value<0.01 (WT vs KO). (B,C) Serum insulin and leptin were measured for WT (grey) and CD38 KO (black) on ND or HFHSD with 6 hrs of fasting. n = 8. ††, p value<0.01 (ND vs HFHSD); **, p value<0.01 (WT vs KO).

HFHSD fed mice develop obesity, hyperleptinemia, hyperglycemia and hyperinsulinemia, compared to normal diet controls (Table 1). A comparison of WT to CD38 KO mice on regular chow diet shows no obvious differences in fasting insulin, free fatty acid, triglyceride, or cholesterol levels. Body weight and fasting glucose are slightly but significantly lower in KO mice (S1 Table). Wild type mice challenged with HFHSD for 4 months gain over 20 g of body weight, whereas the CD38 KO mice gain only about 13 g (Fig 2A). Fat mass accounts for the majority of this difference (Fig 2B), as individual fat depots weight 25%-33% less than WT controls. Muscle weights are similar between both genotypes (Fig 2C), confirming that the body weight differences between genotypes is primarily due to fat pad mass. Both WT controls and CD38 KO mice are equally active (S1 Fig). Surprisingly, CD38 KO mice consume slightly more food per gram body weight compared to WT control (S2 Fig), suggesting that activity and food intake were not responsible for the differences observed on HFHSD. The serum chemistry profiles suggest that CD38 KO mice are less susceptible to the metabolic effects induced by HFHSD and exhibited significantly lower fasting glucose, insulin, and leptin levels than the wild type HFHSD fed controls (Fig 3A–3C), indicating better glycemic control in CD38 KO mice, although it should be noted that these levels are still significantly elevated compared to a normal-chow comparator. Circulating lipids, including free-fatty acid, triglyceride, or cholesterol levels are not different between genotypes on HFHSD (S3 Fig).

**Fig 4:**
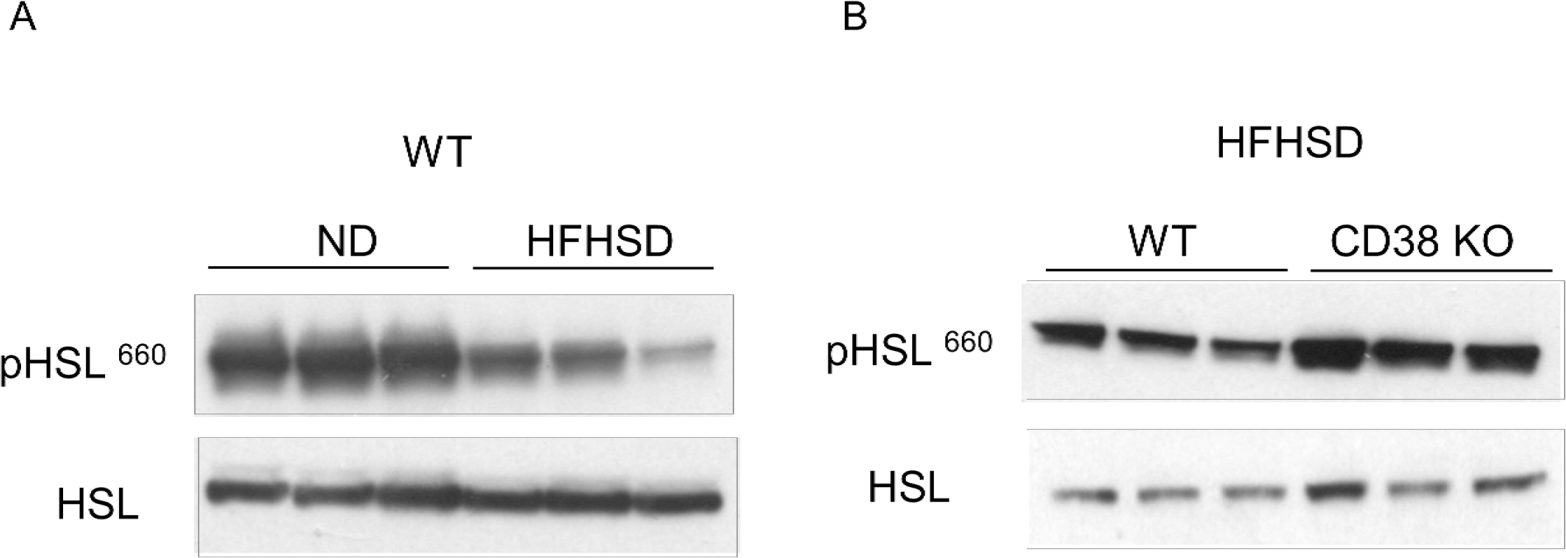
CD38 KO mice retain sensitivity to beta-adrenergic signaling. (A) Lysates from WAT of C57Bl6 mice fed with either ND or HFHSD were immunoblotted with indicated antibodies. (B) Lysates from WAT of WT or CD38 KO mice on HFHSD were immunoblotted with indicated antibodies.

As shown in Fig 2B, the fat mass of CD38 KO mice remained constantly lower compared with WT controls throughout the HFHSD treatment. We asked whether an alteration in adipose physiology may be responsible for the genotype associated difference in adipose tissue mass. Hormone sensitive lipase (*Hsl*) is a key regulator of lipolysis and its enzymatic activity is in turn increased by beta-adrenergic signaling. We observed that phosphorylation of serine 660 of Hsl in white adipose tissue is reduced in the WAT of mice on a HFHSD (Fig 4A).This result indicates that lipolytic pathways are inhibited and this in turn is consistent with the observed adipose mass increase. Interestingly, loss of CD38 preserves this beta-adrenergic receptor signaling in the WAT of mice on the HFHSD (Fig 4B), suggesting that loss of CD38 in adipose tissue can prevent diet-induced catecholamine resistance and therefore facilitate lipolysis and the delivery of fatty acids to tissues for use as fuel.

In order to determine whether the reduced fasting glucose, insulin, and leptin levels in CD38 KO mice (on HFHSD) indicate any physiological impact on whole body insulin sensitivity, oral glucose tolerance and intraperitoneal (IP) insulin tolerance tests were conducted. There are no significant differences between genotypes in the oral glucose tolerance test or insulin tolerance test results (S4A and S4B Fig), in contrast to other reports that did observe such differences [11]. Results similar to ours showing no significant differences were also reported by an independent group [18]. This result implies that the loss of CD38 enzymatic activity and the consequential elevation of tissue NAD^+^ results in improvement of whole-body glycemic control without significantly enhancing peripheral insulin sensitivity in mice.

### CD38 KO mice are protected from western diet-induced exercise intolerance

To determine whether the loss of the CD38 gene beneficially impacts the overall physical fitness of the HFHSD fed mouse we performed an exhaustive exercise test. This test measures exercise capacity and thus is a good indicator of overall physical health. Mice challenged with 5 months of ND or HFHSD were compared via a treadmill running test. This test is designed to run animals to exhaustion. Following an acclimation at a 10° incline, mice were run at 10m/min for 5 minutes. After this 5 minute period, speed is increased by 2m/min every 2 minutes, until exhaustion. The maximum speed used was 40m/min. Mice on HFHSD had gained ~22 g of body weight, and the run time, speed and distance decreased relative to ND controls (Table 2). As mice gain weight, the work load increases in proportion to their body weight, therefore it is necessary to calculate the “work” generated by each mouse. Mice on a HFHSD show a significant decrease of total work performed (21 joule to 16 joule), suggesting chronic HFHSD causes a significant deficit in exercise capacity. 60% HFD also produced a similar qualitative, but not as significant effect.

**Table 2:** Comparison of exercise capacity with different diets

Parameters	ND (n = 13)	60% HFD (n = 10)	HFHSD (n = 12)
**BW (g)**	31.85±0.81	48.96±2.09^††^	53.93±1.98^††^
**Run time (min)**	22.41±0.23	15.68±0.74^††^	12.72±0.92^††^ ^¶^
**Run distance (m)**	390.67±6.85	230.28±14.5^††^	178.49±15.72^††^ ^¶^
**Run speed (m/min)**	26.62±0.37	20.60±0.63^††^	18.0±0.85^††^ ^¶^
**Work (joule)**	21.15±0.72	18.94±2.06	15.85±1.15^††^

††, p value<0.01 (ND vs diet).

¶, p value <0.05 (HFD vs HFHSD)

**Fig 5:**
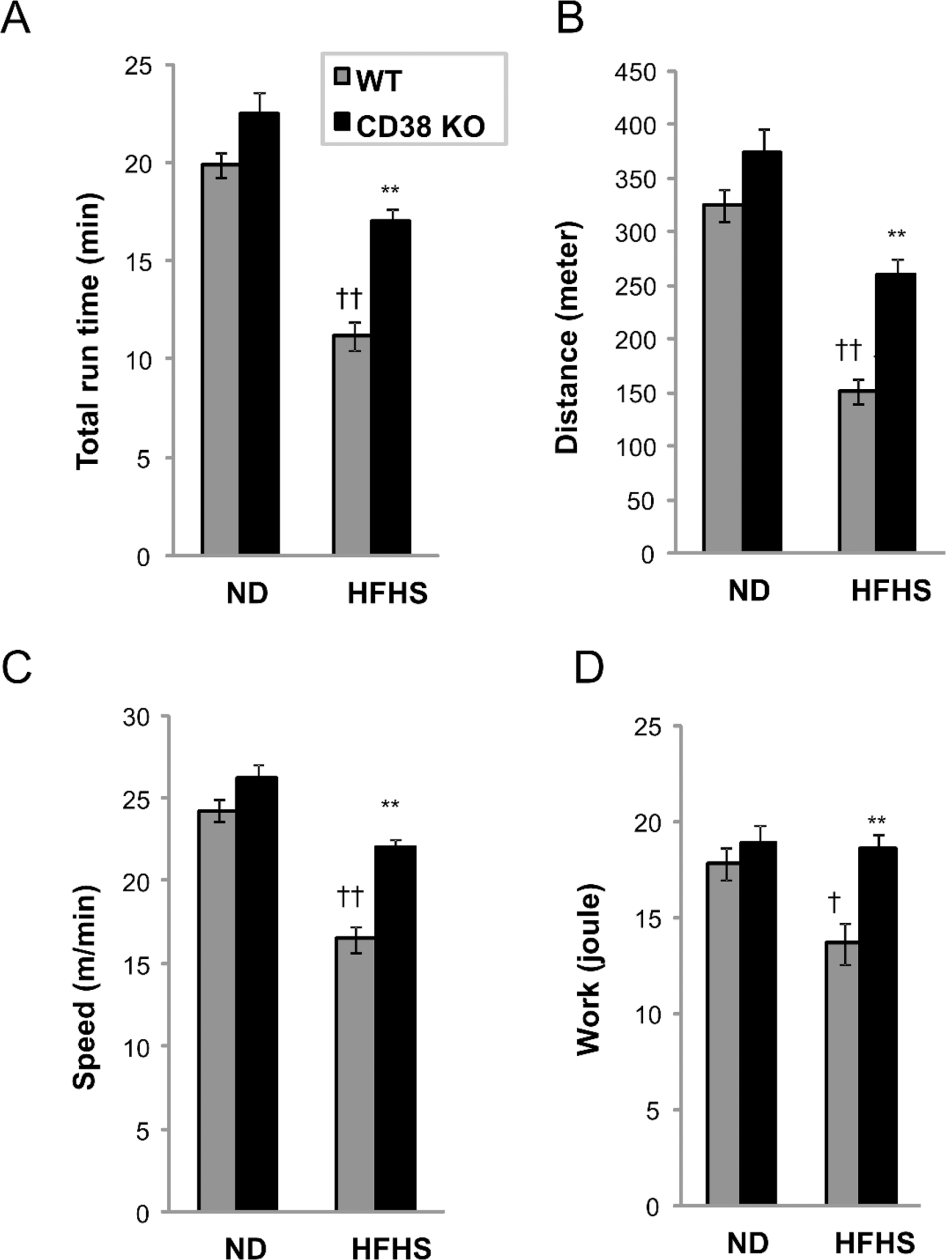
CD38 KO mice are protected from western diet-induced exercise intolerance. (A-D) Treadmill running to exhaustion was performed with WT (grey) and CD38 KO (black) mice fed a ND or HFHSD for 5 months before the study. N = 8 per group. †, p value<0.05 (ND vs HFHS); ††, p value<0.01 (ND vs HFHSD); **, p value<0.01 (WT vs KO). See Material and Methods for experimental details.

We next compared CD38 KO and control mice fed the HFHSD via the treadmill running test. On a normal diet, no differences were observed between WT and CD38 KO (Fig 5A–5D, left bars). HFHSD caused a reduction of 43% in run time, 53% in run distance, 31% of speed and 23% of calculated work (Fig 5A–5D, grey bars). Interestingly, CD38 KO mice show a 52% improvement in run time, 73% improvement in run distance, and 33% improvement of speed compared to WT controls on HFHSD (Fig 5A–5D, right bars). The work performed by KO mice improved 36% which is almost a complete protection of the observed diet-induced deficit (Fig 5D). This result shows that genetic ablation of CD38 protects mice from diet-induced exercise intolerance.

### Genetic ablation of CD38 preserves metabolic flexibility

**Fig 6:**
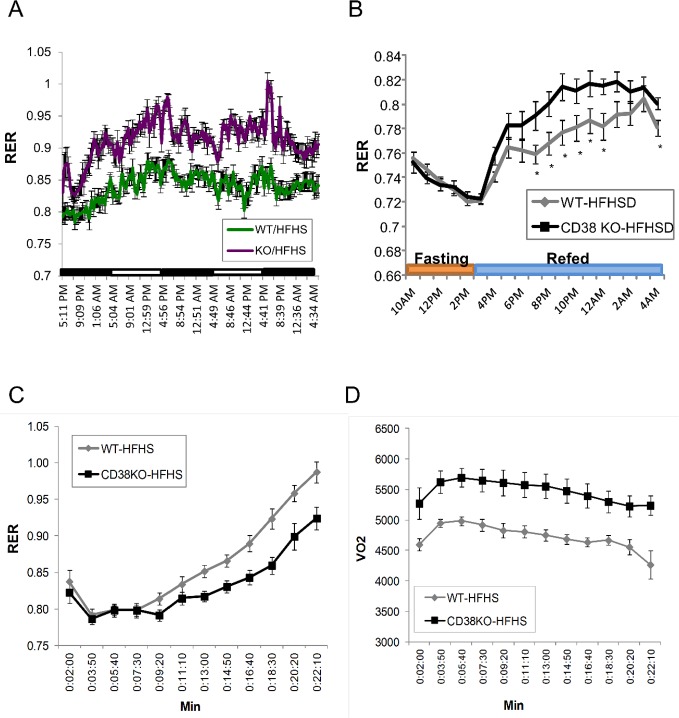
Genetic deletion of CD38 preserved metabolic flexibility. (A) Respiratory exchange ratio (RER) was measured for WT (green) and CD38 KO (purple) mice fed with HFHSD for 2.5 days. N = 8 per group. (B) Respiratory exchange ratio (RER) was measured for WT (grey) and CD38 KO (black) mice fed with HFHSD and fasted for 5 hrs. Food was removed during 10am-5pm. Food was re-supplied at 3pm. N = 12 per group. *, p value<0.05 (WT vs KO). (C) Respiratory exchange ratio (RER) was measured for WT (grey) and CD38 KO (black) mice fed with HFHSD during treadmill running. N = 12 per group. *, p value<0.05 (WT vs KO); **, p value<0.01 (WT vs KO). (D) Oxygen consumption (VO_2_) was measured for WT (grey) and CD38 KO (black) mice fed with HFHSD during treadmill running. N = 12 per group. *, p value<0.05 (WT vs KO); **, p value<0.01 (WT vs KO).

We next sought to determine which physiological parameter best reflects the improved exercise performance in the CD38 KO HFHSD fed mice. One parameter that can impact exercise performance is the balance of carbohydrate to lipids being burned for fuel by the animal. The respiratory quotient (RER = VCO_2_/VO_2_) is thought of as a biochemical measurement of metabolic fuel selection, which is used to quantify the relative oxidation of sugar and lipids. RER peaks when lights are off, and fluctuates between 0.9 and 1.0 in mice on ND for both genotypes (S5 Fig). On HFHSD, RER fluctuates between 0.8 and 0.85 in WT controls, and fluctuates between 0.85 and 0.95 in KO mice (Fig 6A). This result suggests that WT mice mainly use lipids as fuel source at resting state even with high content of both fat and carbohydrates in diet. The higher RER at resting state in CD38 KO mice on HFHSD indicates the preservation of glucose disposal through carbohydrate oxidation.

In order to investigate the role of CD38 gene ablation on metabolic flexibility, RER was compared between control and KO mice that were fasted for 6 hrs followed by re-feeding when the lights were turned off. As shown in Fig 6B, fasting dramatically decreases RER, suggesting that a switch to fat burning occurs in both WT and KO mice. When the HFHSD diet is re-supplied, RER for both treatment groups increases. Interestingly, CD38 KO mice oxidize more carbohydrate immediately after re-feeding and show a higher preference for carbohydrate oxidation throughout the evening, suggesting that the CD38 KO has altered metabolic flexibility in response to nutrients as compared to the WT.

Fuel preference during exercise was compared between WT and CD38 KO mice. During treadmill running to exhaustion, mice burn through fat and carbohydrates based on exercise duration and intensity. WT and CD38 KO mice on chow diet start with a RER around 0.8–0.85. With an increase of running slope and speed, both genotypes consume increasing proportions of carbohydrate with similar RER curves during the treadmill run (S6 Fig). Mice chronically fed the HFHSD mainly use lipids as fuel during the first 10 minutes of low intensity running. Shortly after the speed and slope are increased, muscle starts to oxidize carbohydrate until reaching fatigue at approximately 22 min of run time. CD38 KO mice appear to increase carbohydrate oxidation slightly later then WT mice at 16 min of running and reach fatigue at 28 min of running (Fig 6C). At the end of the run time, both genotypes show an RER = 1 and blood lactate levels above 10mmol/l.

Next we compared oxygen consumption and CO_2_ evolution between two genotypes (S7 Fig). Both O_2_ consumption and CO_2_ production dramatically drop around 21 min of run time in WT mice, whereas CD38 KO mice continue running with a steady VO_2_ and VCO_2_ until 27 minutes of run time. This result suggests that WT mice fatigue earlier as evidenced by increased blood lactate and shorter overall run times. Moreover, the amount of O_2_ consumed in KO mice is consistently higher (12%) than WT controls during the first 20 min of run time (Fig 6D), suggesting that mitochondrial oxidative phosphorylation is more active in the CD38 KO mice during both low and high intensity running. Results under both fasting and exercise conditions consistently suggest that CD38 KO mice are protected from diet-induced metabolic inflexibility.

### Treadmill running induces robust changes in skeletal muscle gene expression independent of genotype

**Fig 7:**
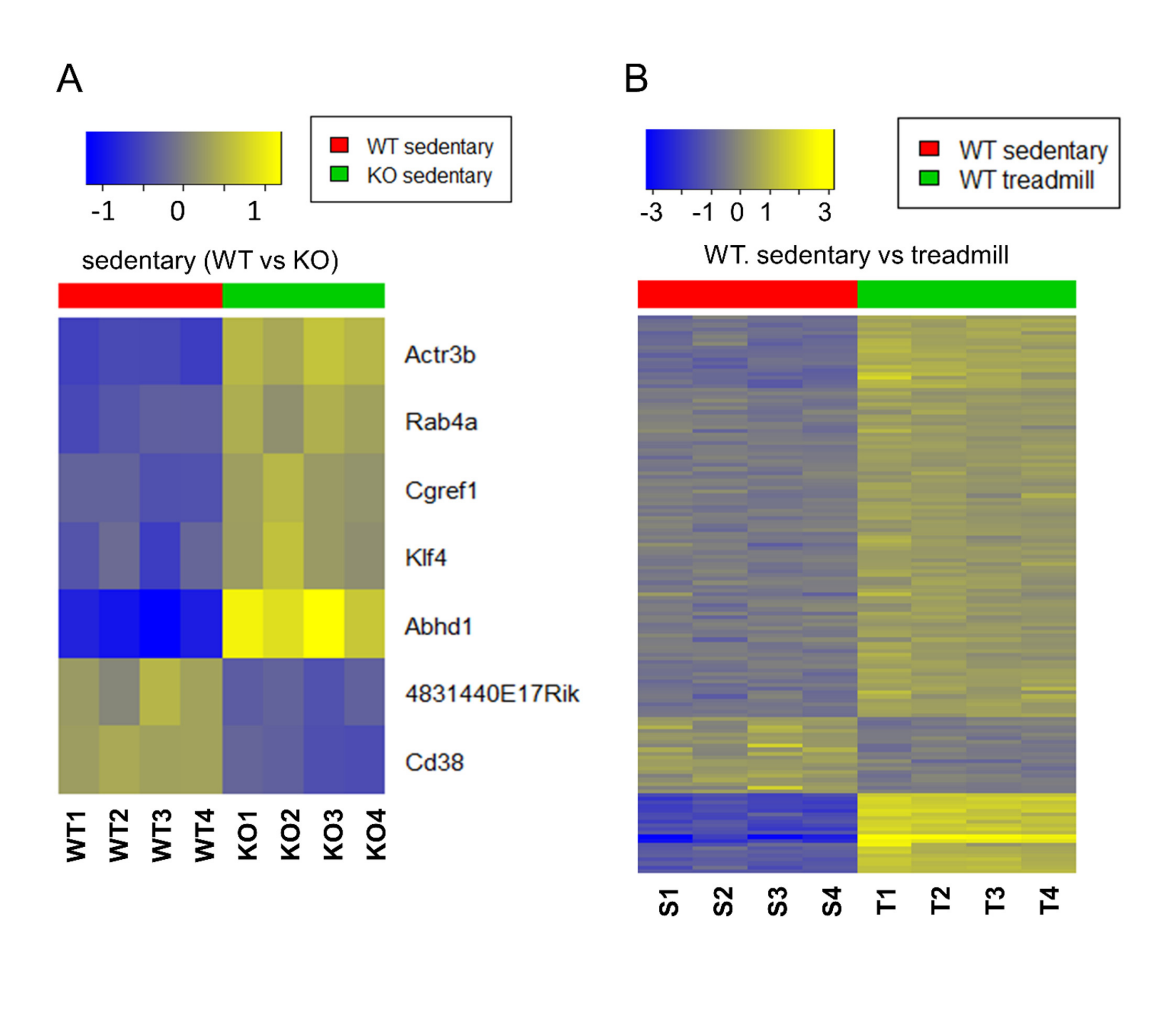
Heat maps showed differential gene expression in CD38 KO mice. (A) Heat maps of gene expression in sedentary samples from gastrocnemius comparing WT (red) and CD38 KO mice (green). Yellow denotes up-, and blue down-regulation. Genes and samples are represented by *rows* and *columns*, respectively. Genes with FDR < 0.1 and absolute fold-change > 1.5 are shown in the heat maps and are clustered by hierarchical clustering (dendrograms not shown). (B) Heat maps of gene expression comparing sedentary (red) vs. treadmill (green) in gastrocnemius of WT samples (right).

We next asked if changes in the response of the transcription program of skeletal muscle to treadmill running might explain the increased endurance of the CD38 KO HFHSD mice. We first compared the transcriptomes of sedentary WT and KO CD38 HFHSD mice to determine whether there are any differences in the rested state. Strikingly, the expression levels of five genes were significantly altered between the genotypes with four increased in the CD38 KO HFHSD muscle and one decreased (Fig 7A). The increased genes were abhydrolase domain containing 1 (*Abhd1*), Rab4a, member Ras oncogene family (*Rab4a*), actin related protein 3b (*Actr3b*), cell growth regulator with EF hand domain 1(*Cgref1*) and Kruppel-like factor 4 (*Klf4*). The one gene reduced in expression is RIKEN cDNA 4831440E17 (4831440E17RIK). Thus there are few perturbations in gene expression due to genotype in the sedentary state.

We next determined the gene expression changes that are triggered by treadmill running and the impact of the CD38 genotype on these changes. After the completion of the run mice were sacrificed and the gastrocnemius muscle harvested for RNA isolation. In the WT HFHSD samples, 147 gene transcripts were significantly altered after the completion of treadmill running comparing with the rested state (Fig 7B). Analysis of the pathways associated with the transcriptional changes highlighted key biological processes likely to be impacted by the exhaustive exercise. Highly overrepresented Gene Ontology (GO) categories suggest that the immediate response to exhaustive exercise in exercise naïve mice is to initiate the development of vascular system changes that may offer physiological adaption to the exercise (S2 Table). In the CD38 KO HFHSD samples, on the other hand, 146 gene transcripts were significantly altered after the completion of treadmill running comparing with the rested state. The 147 and 146 altered gene transcripts in WT and KO HFHSD samples were combined and resulted in 215 unique gene transcripts. The expression heatmap of these 215 gene transcripts demonstrated that the dis-regulation patterns caused by treadmill running of these genes are essentially identical in WT and CD38 KO HFHSD samples (S8 Fig). This suggests that the loss of CD38 has very little impact on the immediate early transcriptional responses in skeletal muscle to exhaustive exercise. Thus, changes in the transcriptome are driven by exercise and not significantly modified by genotype. The complete gene expression microarray data of this study is available in GEO with accession number GSE69062.

## Discussion

The loss of CD38 activity either by genetic deletion or through the use of a small molecule inhibitor can protect mice from diet-induced obesity and insulin resistance. Molecularly, this is thought to be mediated by elevated NAD^+^ levels and a consequent increase in sirtuin deacetylase activity [11, 12]. We demonstrated here that CD38 KO mice on HFHSD displayed lower body weight and a higher respiratory exchange ratio. We did not observe enhanced insulin sensitivity or glucose disposal in contrast with previous report. We report a dramatic improvement in exercise tolerance in the HFHSD fed CD38 KO mice suggesting broadly beneficial effects of the loss of CD38 on whole body exercise tolerance.

Why do CD38 KO mice weigh less on a HFHSD? It is known that high fat diets can induce a state of catecholamine resistance that impairs the mobilization of stored fat by lipolysis [19–21]. We observed that CD38 KO mice on a HFHSD do not develop diet-induced catecholamine resistance (as assessed by phospho-HSL levels) and thus are likely to preserve the capacity to break down triglyceride stored in fat cells via beta-adrenergic receptor signaling. This suggests that one explanation for the reduced size of the fat depot in CD38 KO HFHSD fed mice is a reduced ability to store lipids and increased peripheral utilization of the fat for energy.

Several unique functions of CD38 on regulating diet-induced metabolic inflexibility and exercise intolerance were observed. Healthy skeletal muscle maintains metabolic flexibility allowing a switch between fat and glucose oxidation in response to nutrients (reviewed in [22–24]). Obesity and insulin resistance are associated with an impaired switch from fat to glucose oxidation after a meal [22]. During prolonged submaximal, fixed intensity exercise lipids are consumed preferentially to carbohydrates. The increased reliance on fat oxidation is to presumably delay the consumption of muscle glycogen [23]. Individuals with obesity or type II diabetes exhibit metabolic inflexibility, which can further affect muscle performance [25]. Loss of CD38 preserved glucose oxidation after a meal and prolonged fat oxidation during the exercise test. This may be linked to the apparently higher level of HSL activity in the fat pads of the CD38 KO HFHSD fed mice.

How does CD38 regulate metabolic flexibility? Several publications have linked elevated NAD^+^ levels (achieved by various means) to beneficial changes in mitochondrial protein acetylation status and the acetylation status of the master regulator of mitochondrial biogenesis PPARGC1α (PGC1α). We attempted to identify such changes in the WAT, BAT, liver, and muscle of the various diet and genotype combinations studied here, but were unable to consistently observe any such change. We hypothesized that the skeletal muscle itself may be, at least in part, responsible for driving the improved metabolic flexibility observed in the CD38 KO HFHSD fed mice. Analysis of whole transcriptome changes of the WT and CD38 KO HFHSD fed sedentary mice revealed only a few gene changes. Thus, it is unlikely that an autonomous change in muscle plays a role in the enhanced exercise capacity. As exercise calls on numerous physiological processes to provide adaptation to the increased demand on muscle it may be that numerous multi-tissue effects accumulate and then culminate in an improved exercise capacity. Clearly further studies are warranted to understand the mechanisms by which loss of the CD38 gene alters whole animal physiology.

Protein acetylation has evolved as a conserved regulatory post-translational modification that modulates activities of mitochondrial enzymes and control gluconeogenesis, glycolysis and TCA cycle by histone acetyltransferases (reviewed in [26]). Excessive nutrient supply increases acetyl CoA levels in mitochondria, and increased the global acetylation state of mitochondrial proteins [27]. Changes in NAD^+^ levels have been implicated as a key regulator of the acetylation state of specific target proteins via the NAD^+^ dependent protein deacetylase enzymes known as Sirtuins (reviewed in [28, 29]). We did not observe a consistent correlation between the levels of NAD^+^ in a given tissue and the acetylated state of proteins (S9 Fig). This was the case when we examined the relationship between reduced NAD^+^ levels due to diet and the relationship between elevated NAD^+^ tissue levels and protein acetylation in CD38 KO mice. Thus our results suggest that fluctuating NAD^+^ levels do not explain the changes in the acetylated state of the proteins examined. The data further implies that Sirtuin activity, to the extent that it may be regulated by NAD^+^ flux, plays at best a minor role in regulating protein acetylation in this model.

Finally, while it is clear that the loss of the CD38 gene results in elevated tissue levels of NAD^+^ that are correlated with the physiological effects described here, it is not possible to conclude that loss of the CD38 enzymatic activity and subsequent effects on NAD^+^ levels are solely responsible for these effects. CD38 catalyzes the conversion of NAD^+^ to the second messenger molecules cyclic ADP-ribose (cADPR), ADPR, and nicotinic acid adenine dinucleotide phosphate (NAADP). It is possible that reduced levels of the products of CD38 enzyme activity, and not elevated NAD^+^, are mediating some or all of the effects observed here. Alternatively, some other function of CD38 may be responsible for the observed phenotypes, such as its proposed function in cell adhesion. The development of a CD38 enzyme dead knockout mouse or highly selective and potent pharmacological inhibitors of the CD38 enzyme activity will facilitate the discovery of the precise role of the enzymatic activity in regulating exercise tolerance in the face of diet induced metabolic dysfunction.

## Material and Methods

### Animals and animal care

All procedures performed were in compliance with the Animal Welfare Act, United States Department of Agriculture (USDA) regulations and approved by the GlaxoSmithKline Institutional Animal Care and Use Committee. Animals were housed in a specific pathogen-free facility at 72°F with a 12-hour light and dark cycle and given free access to food and water.

Cohorts of male C57Bl/6J and CD38 KO mice were purchased from the Jackson Laboratory (Bar Harbor, ME) and housed in the GlaxoSmithKline vivarium. The mice were individually housed in standard shoebox cages and allowed free access to water. Beginning at 8–10 weeks of age, C57Bl/6J and CD38 KO mice were assigned to one of the three groups and fed a standard diet (ND, Lab Diet 5001, Purina Mills Inc.), a high fat diet consisting of 60% of calories from fat (HFD, D12492 Research Diets Inc.) or high-fat, high-sucrose diet (HFHSD, D12331 Research Diet) for 14–18 weeks.

While in the vivarium, body weight and body composition of each mouse was monitored at regular intervals. Prior to the exercise protocol all animals were placed in the Columbus Instruments 8-lane modular treadmill to acclimate to the environment and belt movement. Unless mentioned in the figure legends, most of the experiments were performed 6 hours after withdrawal of food.

### Reagents

All chemicals were obtained from Sigma-Aldrich unless stated otherwise. Enhanced chemiluminesence (ECL) reagents were purchased from NEN, Inc. EDTA-free protease inhibitor tablet was purchased from Roche, Inc. Anti-Acetylated lysine, anti-Ac-p65 and anti-Foxo1 were purchased from Cell Signaling. Anti-Ac-Foxo1/3 was obtained from Santa Cruz Biotechnology.

### Whole blood and plasma measurements

Whole blood was collected into heparin tubes. Plasma insulin concentrations were measured by insulin ELISA kit (Crystal Chem Inc.). Blood glucose was measured by OneTouch Ultra Glucometer. NEFA, cholesterol Triglyceride were measure Olympus AU460 Automated Chemistry Immuno System. Adiponectin, and leptin, MCP-1, TNF-α were measured by adipokine panel (Millipore EMD).

### Glucose and insulin tolerance tests

To determine the oral glucose tolerance of each group of mice, animals were first fast for 6hrs, and administered a glucose solution (2g/Kg body weight) by oral gavage. Blood glucose was determined from a tail nick at basal (t = 0) and times indicated in figures using a One Touch Ultra glucometer (Lifescan). For insulin tolerance tests, mice were given an intraperitoneal injection of 1 unit insulin/kg body weight after 3 hrs of fasting. Blood glucose concentrations were determined as described above.

### Blood and tissue NAD^+^ measurement

Blood samples were collected and diluted 1:1 with water containing 5% EDTA and snap frozen. Blood samples were removed from the freezer and immediately diluted 1:4 with 80:20 acetonitrile: water that contained O^18^ labeled NAD^+^ and excess EDTA. All tissue samples were snap frozen in liquid nitrogen to minimize the degradation of NAD^+^ during sample collection. Samples were kept on dry ice and diluted 1:4 with 80:20 acetonitrile:water that contained O^18^-labeled NAD^+^ and excess EDTA and an inhibitor of CD38 enzyme. Samples were immediately homogenized in a bead beater (MP FastPrep) with metal beads for 60 sec at 6 m/sec. (2 x 60 sec for gastroc) and then centrifuged 5 minutes at 13,000 rpm. The sample extract was diluted 1:10 with water and 10 μL injected on a Zorbax Hillic Plus column on an Agilent 1290 HPLC and a Sciex API4000 Mass Spectrometer monitoring the 664–428 transition for NAD^+^ and 668–136 for O^18^-NAD internal standard. Results reported as normalized based on the area ratio of NAD to the spiked O^18^-NAD internal standard to correct the variable rate of degradation of NAD in various tissue matrixes. The LC separation was achieved with Mobile Phase A—Water with 0.1% Ammonium Acetate and Mobile Phase B—Acetonitrile w/ 0.1%formic acid using the following gradient (Table 3).

**Table 3:** Blood and NAD^+^ measurement.

Step	Total Time (min)	Flow rate (μl/min)	A (%)	B (%)
0	0	600	2	98
1	0.5	600	2	98
2	2.5	600	80	20
3	2.9	600	80	20
4	3	600	2	98

### Protein lysates and western blot analysis

Frozen tissues were homogenized on ice in lysis buffer (50mM Tris, pH7.5, 5mM EDTA, 250mM sucrose, 1% NP40, 2mM DTT, 1mM sodium vanadate, 100mM NaF, 10mM Na_4_P_2_O_7_, and freshly added protease inhibitor tablet), ground and rocked for 1 hr in cold room [30]. Crude lysates were then centrifuged at 14,000 x g for 15 minutes twice and the protein concentration was determined using BioRad Protein Assay Reagent. 30 μg proteins were resolved by a 4–12% or 12% NUPAGE Bis-Tris precast gel electrophoresis and transferred to nitrocellulose membranes (Invitrogen). Individual proteins were detected with the specific antibodies and visualized on film using horseradish peroxidase-conjugated secondary antibodies (BioRad) and Western Lightning Enhanced Chemiluminescence (Perkin Elmer Life Sciences).

### RNA Isolation and Gene Expression

Total RNA was isolated from flash-frozen mouse tissue samples by using Trizol (Invitrogen) isolation method and cleaned up with RNAEasy mini columns (Qiagen). cDNA was produced using a high capacity cDNA transcription kit (Applied Biosciences). The primer probe sets were ordered from IDT (Integrated DNA Technologies). The quantitative expression of each gene was assessed using Taqman Gene Expression Assays on an Applied Biosystems 7900HT machine. Relative expression was calculated using the delta-delta C_t_ method.

### CLAMS

Indirect open circuit calorimeter was performed using the Comprehensive Lab Animal Monitoring System (CLAMS; Columbus Instruments, Columbus, OH). In each experiment, thirty two mice were weighed before testing. Each mouse was placed in its own chamber without bedding and allowed to acclimate to its surroundings; mice were not pre-acclimated to the CLAMS cages prior to the experiment. All experiments began at 9–10 am and continued for up to 48 hrs. Phase 1 of the study assessed metabolic preference during a non-feeding state; food was removed from the cages (10AM–4PM). Phase 2 evaluated metabolic preference during a feeding condition; food in excess was provided at 4 PM. The system measured oxygen consumption, carbon dioxide production and calculated the Respiratory Exchange Ratio (*RER = VCO2/VO2*) for each animal. Energy expenditure was also estimated using the formulas:

CaloricValue(CV)=3.815+(1.232*RER)EnergyExpenditure(EE)=CV*VO2

Activity was determined using an array of infrared beams (2.5 cm inter-beam distance) surround each cage. Total activity was monitored continuously and any movement that produced a beam break was counted and summed.

### Treadmill running

Exercise endurance was determined using a Columbus Instruments modular treadmill. Eight mice could be run at a time in this system. Prior to the measurement, all mice were weighed and allowed to orient to the inclined treadmill (10°) surroundings for 3–5 minutes. A ramping program was then begun at 4 m/min for 5 min to allow the animals to acclimate to the movement of the belt and orient them to the shock grid. The speed was increased to 10 m/min for 10 min and then increased 2 m/min every 2 minutes (to a maximum speed of 40 m/min) until the animals run to exhaustion. Exhaustion was defined by an RER ~1.0 and the unwillingness to move from the shock grid after 6 shocks. Run time, run distance, work performed, VO_2_, VCO_2_, respiratory exchange rate (RER) were determined during each bout on the treadmill.

Respiratory Exchange Ratio (RER) during both the CLAMs and treadmill exercise protocols was calculated as *RER = VCO*_*2*_ */VO*_*2*_. Under normal circumstances, an RER value of 0.70 indicates that fat is the predominant fuel source; an RER of 0.85 suggests a mix of fat and carbohydrates, and a value of 1.0 or above suggests that carbohydrates are the predominant fuel source. In our exercise protocols, an RER > than 1.0 served as a secondary endpoint criterion for exhaustion.

Work performed during each exercise bout was calculated as follows:

$$Work\;\left(joule\right)\;=\;Mass*\;Distance*\text{sine of angle of incline}=\;BW\;\left(kg\right)\;*\;Distance\;Run\;\left(m\right)\;*\;sin10^{o}$$

### Statistical analysis

Averaged values are presented as the mean ± s.e.m. When comparing two groups, we determined statistical significance using the student’s t-test. The values for run time, run distance, work performed and RER are expressed as mean ± standard error of the mean. Comparison of WT and KO mice were made using the JMP statistical package (SAS Inc., Cary NC) using a Two-Way Analysis of Variance (ANOVA) followed by Dunnett's post-hoc test. Values were considered to be significant when a value of p<0.05 was achieved

### Microarray data analysis

Gene expression profiles were obtained from Affymetrics GeneChip Mouse Genome 430 2.0 Arrays. The microarray data was first preprocessed, including background correction and normalization, by Robust Multi-array Average (RMA) [31] with custom CDF downloaded from BrainArray [32]. Array quality was assessed using Array Quality Metrics package [33] from Bioconductor. Differential expression analysis was performed using limma package [34] from Bioconductor. To identify genes that were differentially expressed in CD38 KO vs. WT, or exercise vs. sedentary, the fold change and associated statistical significance were estimated by a linear model in each comparison for each gene. The resulted p-value for differential expression was corrected for multiple hypotheses testing using Benjamini & Hochberg’s method (fdr) [35]. Genes with fdr corrected p-value < 0.1 and absolute fold change > 1.5 were selected as differentially expressed genes. The microarray analysis was performed using R statistical software.

Microarray data: **GEO accession number GSE69062**

## Supporting Information

S1 Fig X-Y axis total velocity and activity were measured on mice fed with ND or HFHSD by CLAMS cages. (TIF)

S2 Fig Food intake was measured by an average of food consumption from 3 consecutive days. The amount of food consumed was normalized with body weight. **, p value<0.01 (n = 12).
(TIF)

S3 Fig Blood lipid profile was measured for WT (grey) and CD38 KO (black) after 6 hrs of fasting. n = 8. ††, p value<0.01 (ND vs HFHSD).
(TIF)

S4 Fig (A) Oral glucose tolerance test was performed by fasting mice for 6hrs before orally gavaged with 2g/Kg of glucose. WT (grey) and CD38 KO (black) fed with HFHSD for 5m. n = 11–12 per group. *, p value<0.05; **, p value<0.01. AUC was calculated by area under the curve. (B) Insulin tolerance test was measured by fasting mice for 3hrs before 1U/Kg insulin injection. WT (grey) and CD38 KO (black) fed with HFHSD for 5m. n = 11–12.
(TIF)

S5 Fig Respiratory exchange ratio (RER) was measured for WT (blue) and CD38 KO (red) mice fed with ND for 2.5 days. N = 8 per group.
(TIF)

S6 Fig Respiratory exchange ratio (RER) was measured for WT (dashed grey) and CD38 KO (dashed black) mice fed with ND during treadmill running. n = 12 per group.
(TIF)

S7 Fig Respiratory quotient (RQ, red), oxygen consumption (VO_2,_ green) and CO_2_ production (VCO_2,_ black) was measured on mice fed with HFHSD during treadmill running. n = 12 per group.
(TIF)

S8 Fig Heat maps of differentially expressed genes which show significant response to exercise in either WT or KO samples in sedentary vs. treadmill condition. Yellow denotes up-, and blue down-regulation. Genes and samples are represented by *rows* and *columns*, respectively. Genes with FDR < 0.1 and absolute fold-change > 1.5 included in the heat maps and are clustered by hierarchical clustering (dendrograms not shown).
(TIF)

S9 Fig Loss of CD38 showed minimal effect on acetylated state in gastrocnemias. (A) Lysates from gastrocnemias of C57Bl6 mice fed with either ND or HFHSD were immunoblotted with indicated antibodies. (B) Lysates from Gastroc. Of WT or CD38KO on HFHSD were immunoblotted with antibodies indicated.
(TIF)

S1 Table Body weight and serum profile in WT and CD38 KO mice fed with ND. (TIF)

S2 Table Pathway analysis of genes differentially expressed in gastrocnemias of WT and CD38 KO mice in response to exhaustive exercise. (TIF)
