# Supplementary figures and images for: Genetic Ablation of CD38 Protects against Western Diet-Induced Exercise Intolerance and Metabolic Inflexibility
Source: PLoS One. 2015 Aug 19;10(8):e0134927. doi: 10.1371/journal.pone.0134927 (PMC4546114; doi:10.1371/journal.pone.0134927)

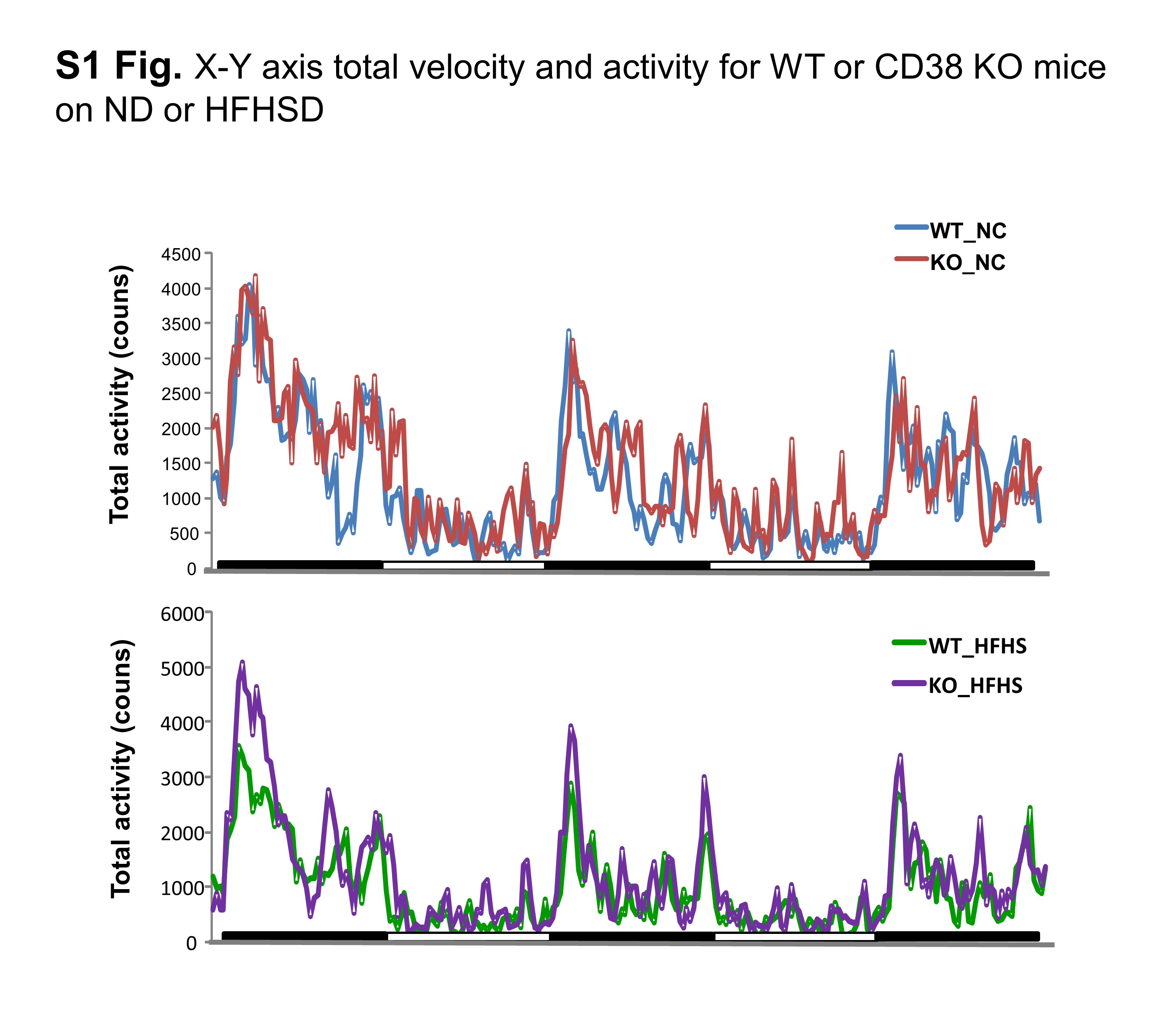

Supplement: S1 Fig — (TIF) [file pone.0134927.s001.tif]

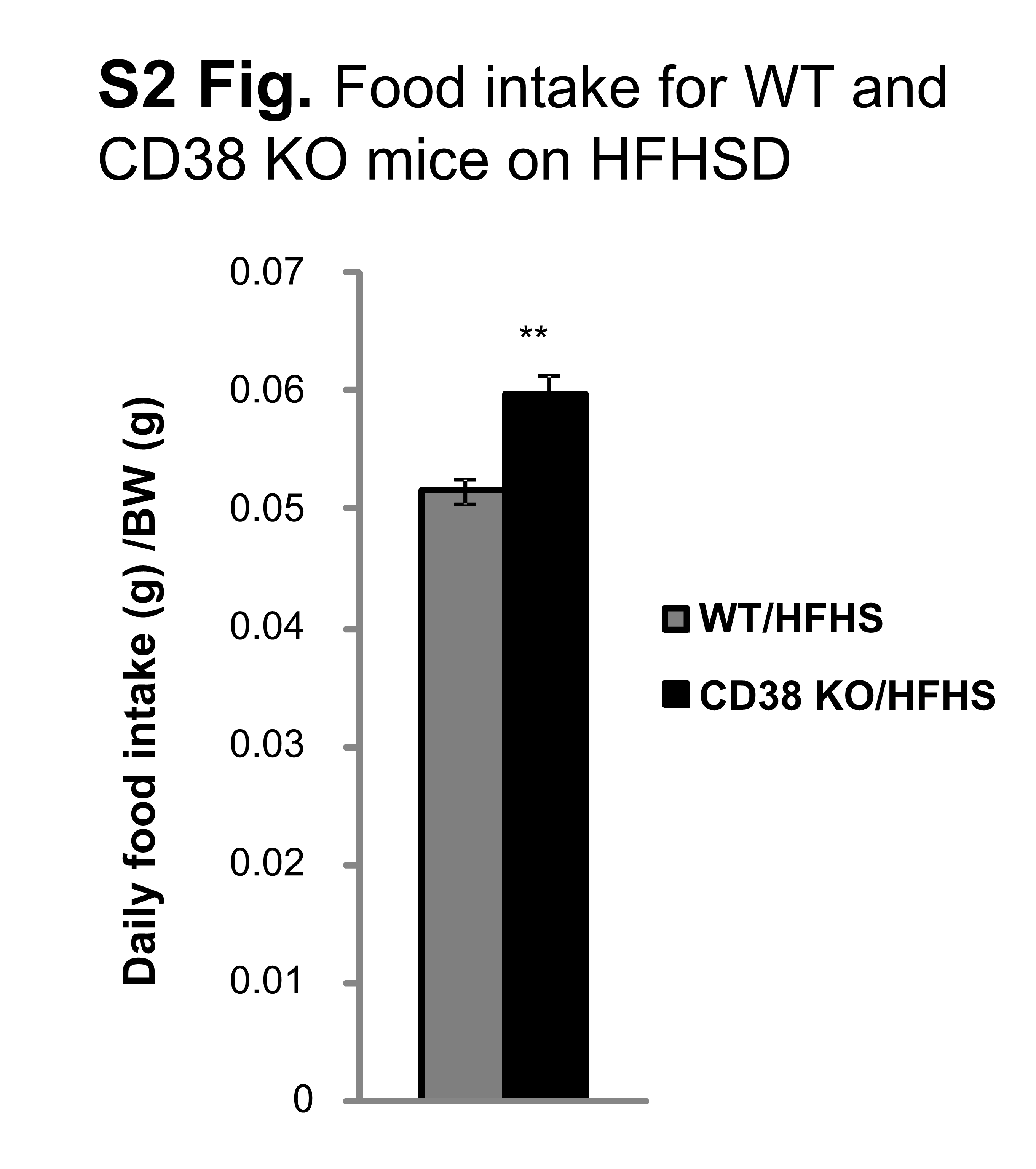

Supplement: S2 Fig — The amount of food consumed was normalized with body weight. **, p value<0.01 (n = 12). (TIF) [file pone.0134927.s002.tif]

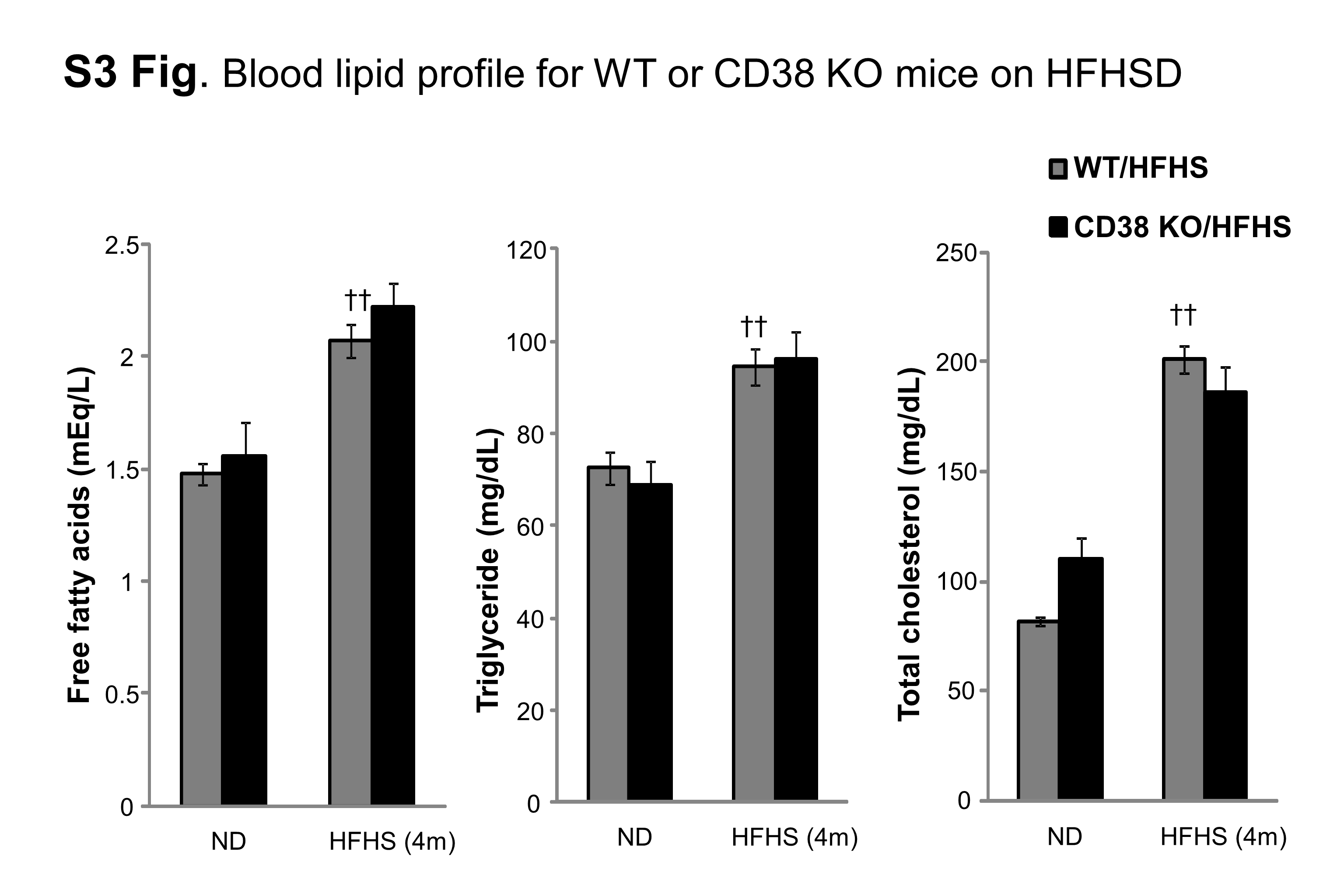

Supplement: S3 Fig — n = 8. ††, p value<0.01 (ND vs HFHSD). (TIF) [file pone.0134927.s003.tif]

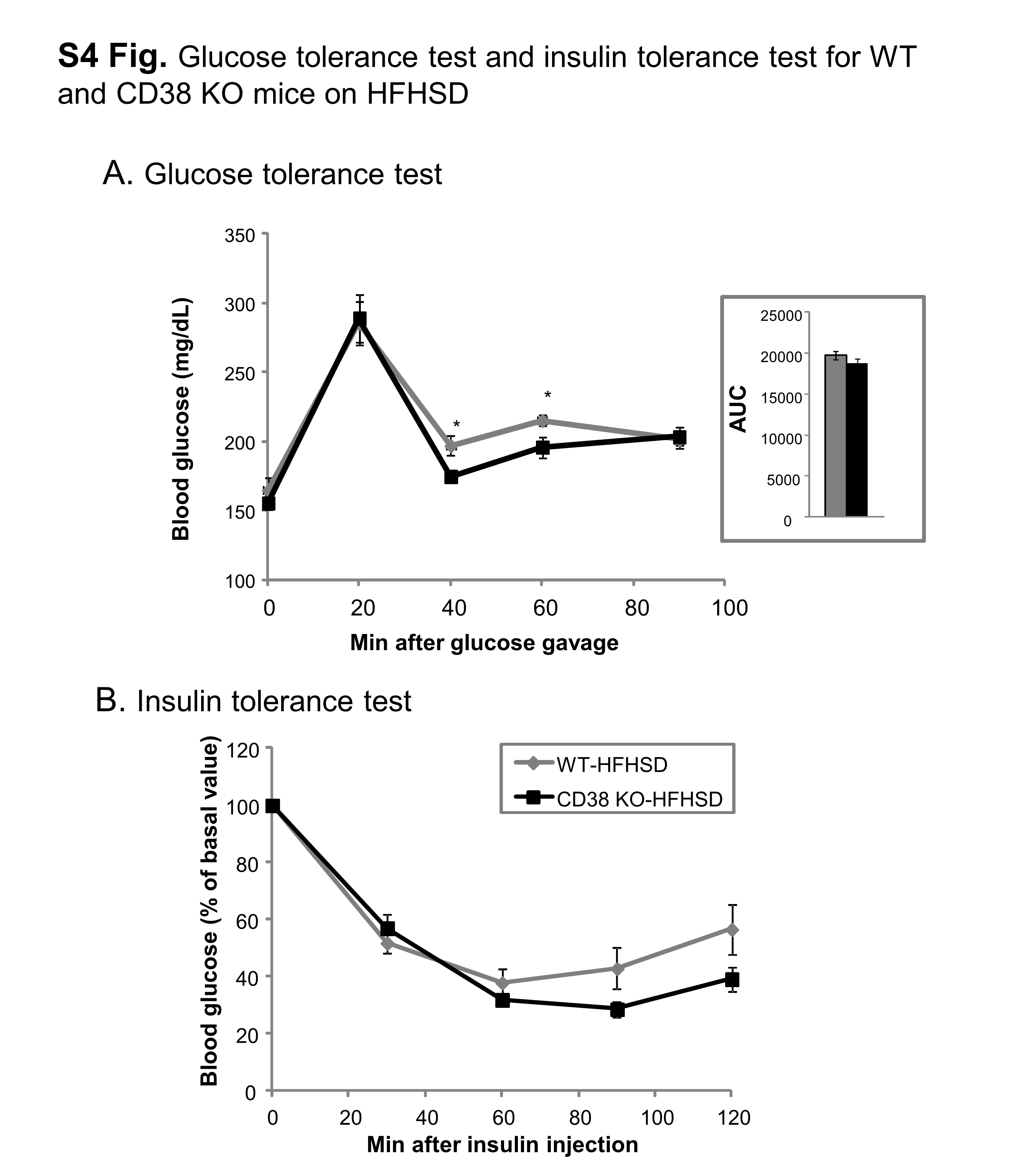

Supplement: S4 Fig — (A) Oral glucose tolerance test was performed by fasting mice for 6hrs before orally gavaged with 2g/Kg of glucose. WT (grey) and CD38 KO (black) fed with HFHSD for 5m. n = 11–12 per group. *, p value<0.05; **, p value<0.01. AUC was calculated by area under the curve. (B) Insulin tolerance test was measured by fasting mice for 3hrs before 1U/Kg insulin injection. WT (grey) and CD38 KO (black) fed with HFHSD for 5m. n = 11–12. (TIF) [file pone.0134927.s004.tif]

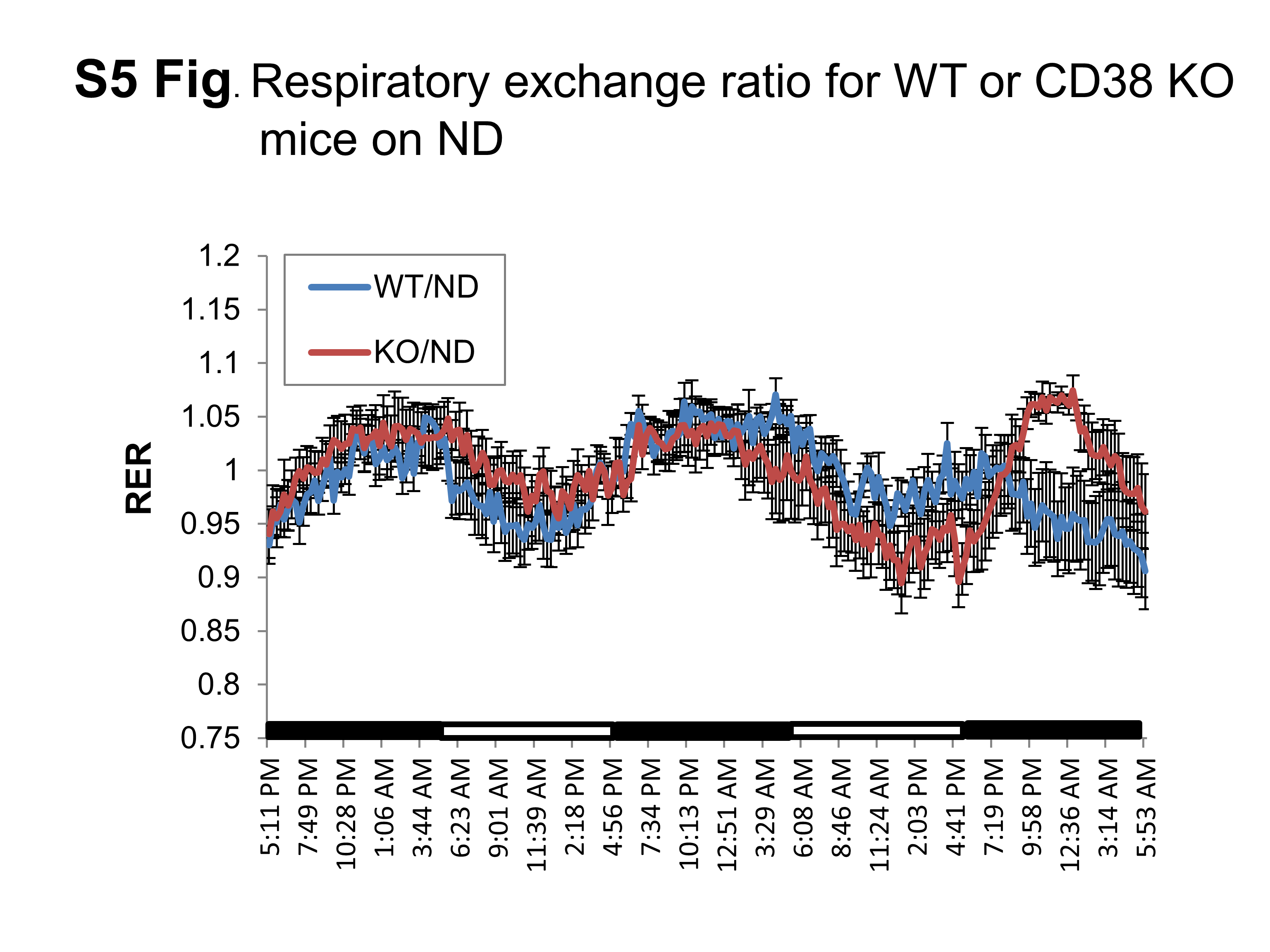

Supplement: S5 Fig — N = 8 per group. (TIF) [file pone.0134927.s005.tif]

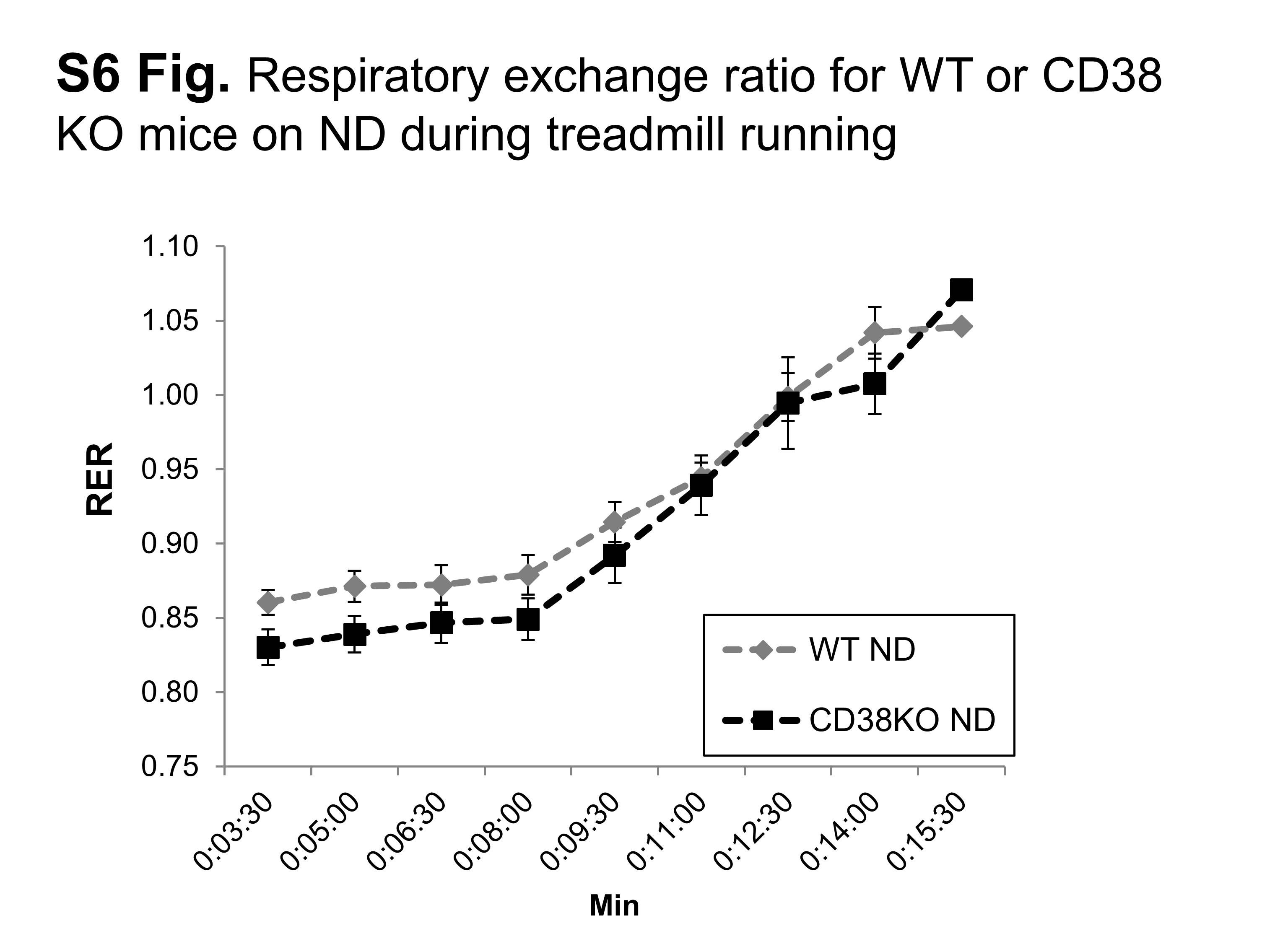

Supplement: S6 Fig — n = 12 per group. (TIF) [file pone.0134927.s006.tif]

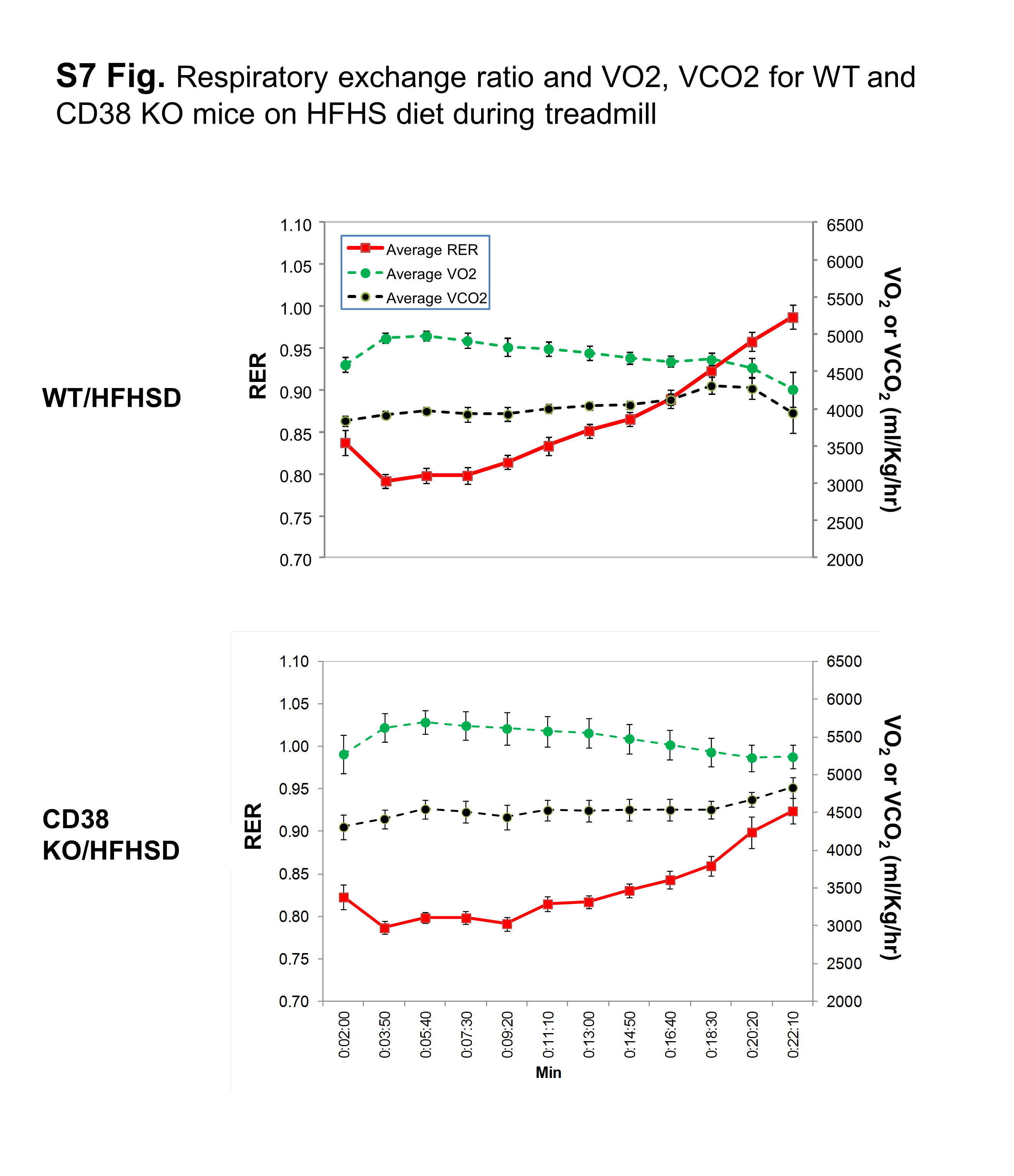

Supplement: S7 Fig — n = 12 per group. (TIF) [file pone.0134927.s007.tif]

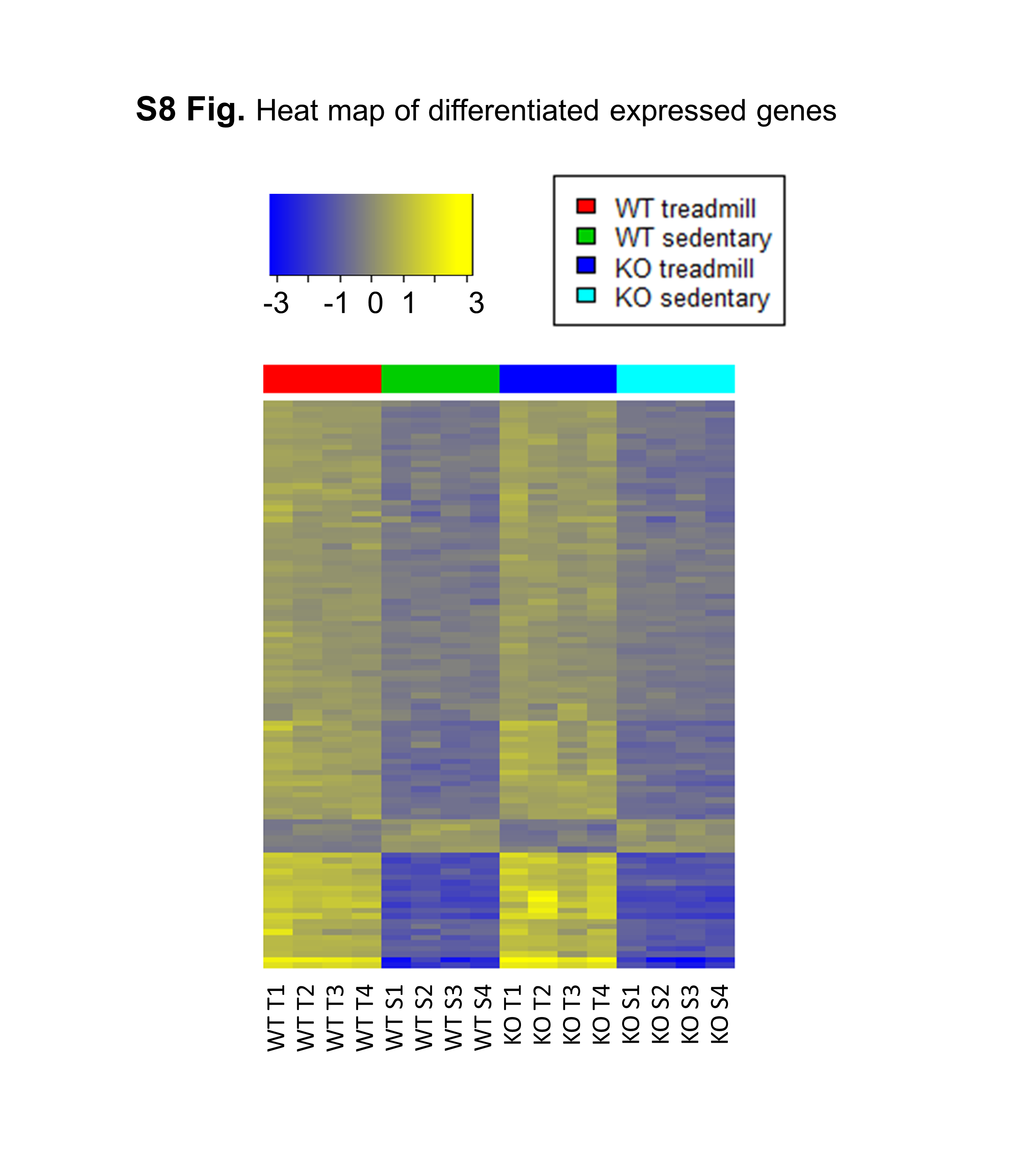

Supplement: S8 Fig — Yellow denotes up-, and blue down-regulation. Genes and samples are represented by rows and columns, respectively. Genes with FDR < 0.1 and absolute fold-change > 1.5 included in the heat maps and are clustered by hierarchical clustering (dendrograms not shown). (TIF) [file pone.0134927.s008.tif]

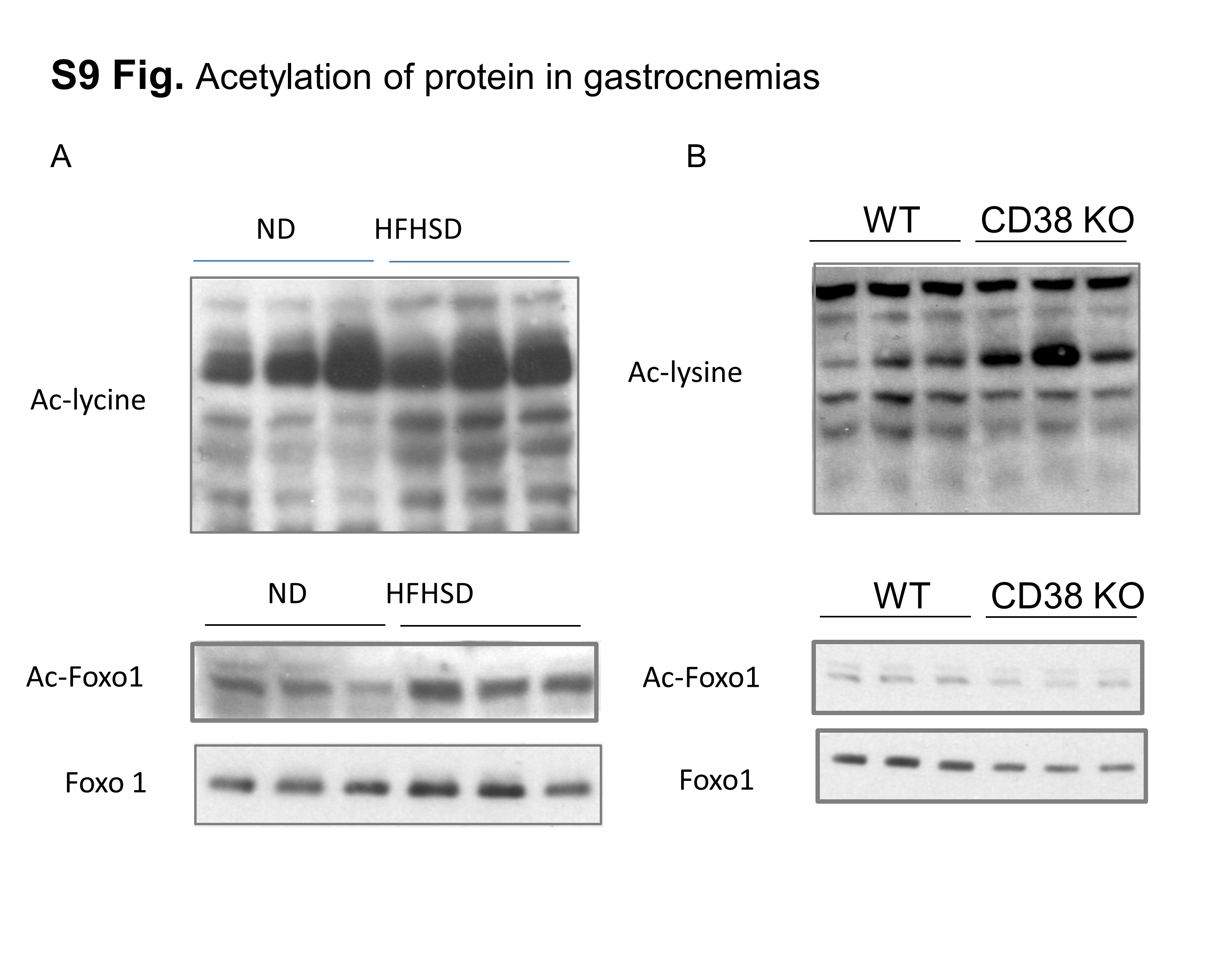

Supplement: S9 Fig — (A) Lysates from gastrocnemias of C57Bl6 mice fed with either ND or HFHSD were immunoblotted with indicated antibodies. (B) Lysates from Gastroc. Of WT or CD38KO on HFHSD were immunoblotted with antibodies indicated. (TIF) [file pone.0134927.s009.tif]

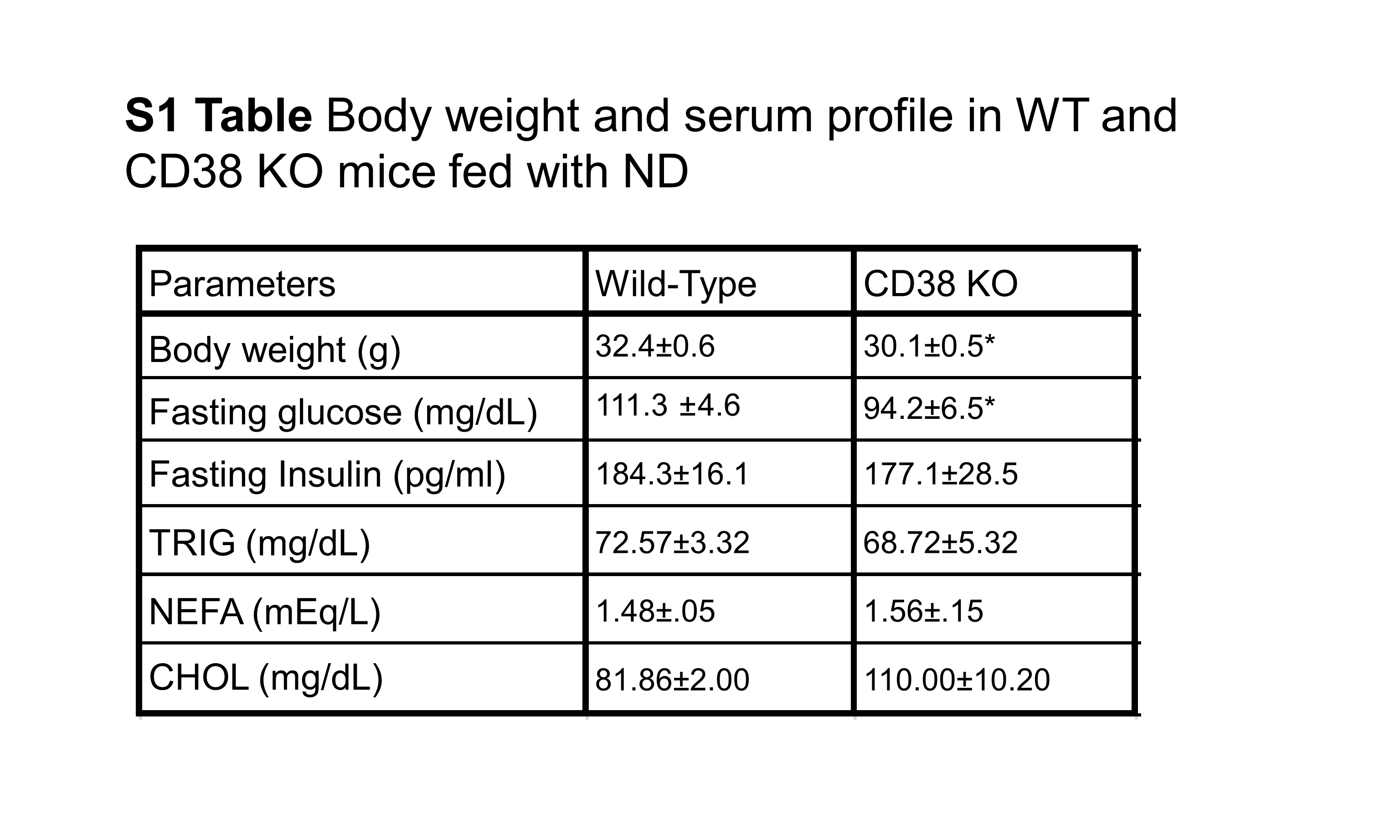

Supplement: S1 Table — (TIF) [file pone.0134927.s010.tif]

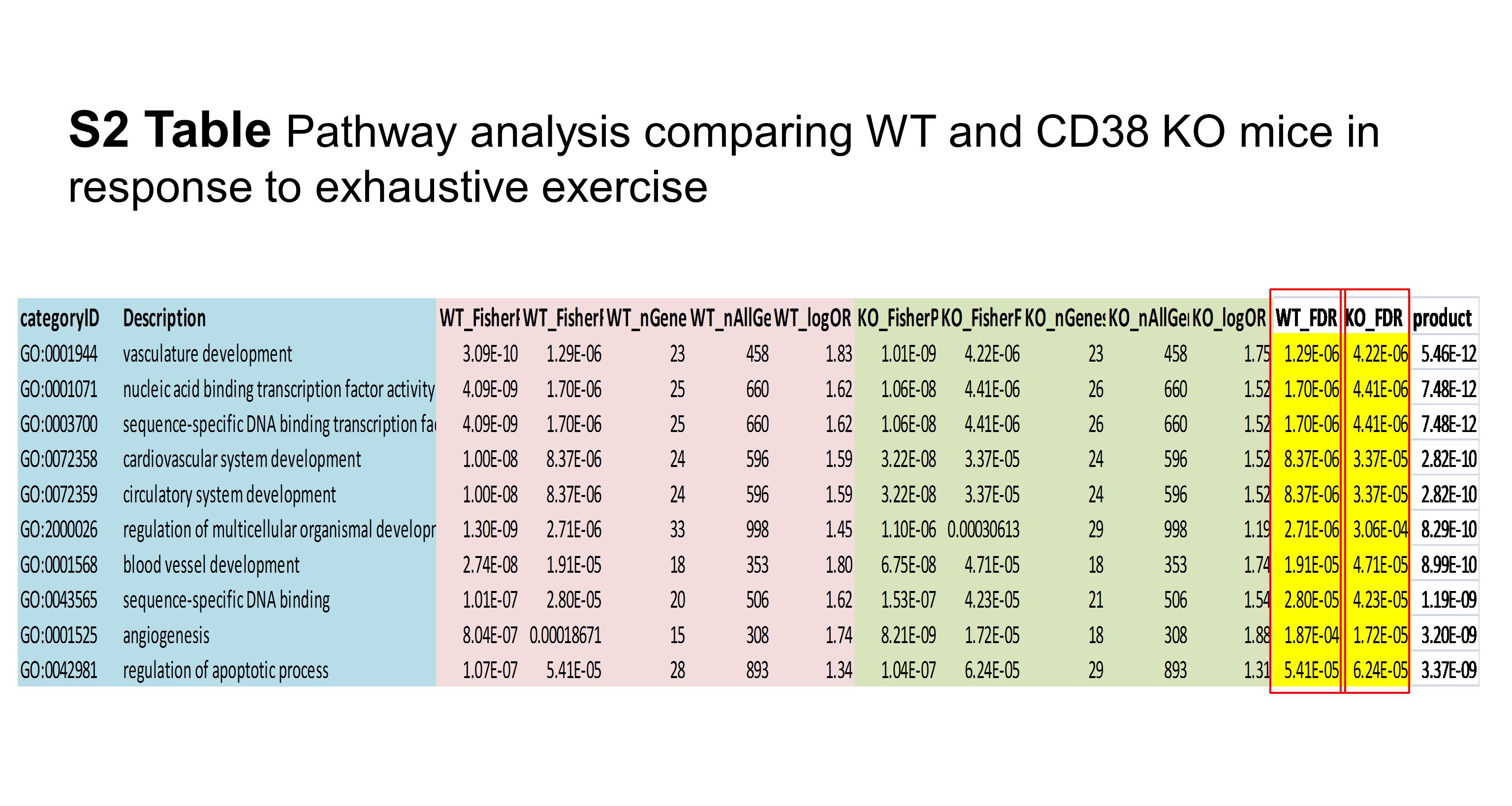

Supplement: S2 Table — (TIF) [file pone.0134927.s011.tif]
